# Identification of the functional domains of canine tetherin in antiviral activity against canine influenza virus

**DOI:** 10.3389/fvets.2025.1560273

**Published:** 2025-05-21

**Authors:** Jiajun Ou, Yixin Dai, Yujie Jiang, Lingzhi Dai, Liang Xu, Gang Lu, Gaoming Lou, Shoujun Li

**Affiliations:** 1 College of Veterinary Medicine, South China Agricultural University, Guangzhou, China; 2 Henry Fok School of Biology and Agriculture, Shaoguan University, Shaoguan, China; 3 Guangdong Provincial Key Laboratory of Utilization and Conservation of Food and Medicinal Resources in Northern Region, Shaoguan University, Shaoguan, China

**Keywords:** tetherin, canine influenza virus, antiviral activity, functional domain, influenza A virus

## Abstract

Canine influenza virus (CIV) is a respiratory pathogen that causes fever, coughing, and sneezing in dogs and is continuously circulating in canine populations. Tetherin is an antiviral host restriction factor mediated by interferon, capable of inhibiting the release of enveloped viruses from infected cells. The antiviral mechanism of tetherin is mainly due to its unusual topology, which includes a short N-terminal cytoplasmic tail (CT), a transmembrane (TM) domain, a coiled-coil extra-cellular region (CC), and a C-terminal glycosyl-phosphatidylinositol anchor (GPI). Previous studies have found that canine tetherin has the ability to limit the release of CIV, but its main antiviral domain remains unclear. In the present study, the potential CT, TM, CC, and GPI domains of canine tetherin were predicted through systemic bioinformatic analysis, and mutational variants of canine tetherin based on the four domains were constructed. Confocal microscopy demonstrated that the CT, TM, and CC domains are critical for the cell membrane localization of canine tetherin. The results of *in vitro* CIV infection experiments showed that the TM region is a critical functional domain of canine tetherin in limiting the replication of CIV. Our study will help better understand the antiviral activity of canine tetherin and the role of the structural domains of canine tetherin in inhibiting the replication of CIV.

## 1 Introduction

Antiviral restriction factors are an integral part of the host’s innate immune system that protects cells from viral pathogens. Upon viral infection, the body induces the expression of type I interferons, which stimulate the expression of many genes that encode innate immune factors, thereby limiting viral replication. As an important member, tetherin (also known as CD317, BST-2, or HM1.24) is an interferon-inducible type II transmembrane (TM) protein with a short N-terminal cytoplasmic tail (CT), a transmembrane (TM) domain, a coiled-coil extracellular region (CC), and a C-terminal glycosyl-phosphatidylinositol anchor (GPI) (1, 2). Tetherin forms homodimers and does not require interaction with specific viral components, except for the viral membrane, which inhibits the release of enveloped viruses, including human immunodeficiency virus type 1 (HIV-1), vesicular stomatitis virus (VSV), dengue virus (DENV), and influenza A virus (IAV) (3–5).

Tetherin has been identified in various species, including dogs, horses, cats, non-human primates, chickens, and rodents (6–11). Despite significant sequence differences, most tetherins from different sources exhibit similar antiviral functions by directly and physically retaining viral particles on the cell surface (12).

Canine influenza virus (CIV), which is responsible for canine influenza (CI), is a respiratory pathogen that can cause clinical symptoms such as coughing, sneezing, fever, and a runny nose in dogs. Affected dogs often develop secondary infections from other pathogens, which can result in respiratory failure and, in severe cases, death (13).CIV was first reported in the USA in 2004, and two subtypes of CIV are currently prevalent: H3N8 CIV, which originated in horses, and H3N2 CIV, which originated in birds (14). Since then, related epidemics have appeared in China, Korea, Thailand, Germany, the UK, and Australia, seriously threatening the health of dogs (15–20).

IAV has the ability to cross species barriers and can transmit from one host to another. To overcome these barriers, the virus requires several adaptations to the new host. Tetherin may act as a barrier to limit the spread of IAV. Currently, research on tetherin-restricted IAV mainly focuses on human tetherin, and this restriction is strain-specific. As one of the hosts of IAV, dogs are potential “mixing vessels.” Therefore, it is important to conduct research on canine innate immune factors against influenza virus. Recent studies have shown that the TM and GPI regions of human tetherin are the main functional domains of its antiviral activity (21, 22). However, the domain that plays a crucial role in the antiviral activity of canine tetherin remains unclear. In this study, we constructed eukaryotic expression plasmids of canine tetherin and its mutational variants with deletions of the canine tetherin structural domains, verified their subcellular localization in cells, and assessed their antiviral activity against CIV.

## 2 Materials and methods

### 2.1 Plasmid DNA, virus, and antibodies

The pEF6/Myc-His vector was used as the eukaryotic expression vector. The coding sequences of canine tetherin (GenBank accession No. XM_038428239) and its mutational variants (delCT and delGPI) were inserted between the KpnI and EcoRI sites of the multiple cloning site, with the Kozak sequence (GCCACC) and a FLAG-tag added to the N-terminus. The eukaryotic expression vector of the canine tetherin mutational variants delCC and delTM were generated using fusion PCR.

Stocks of the CIV strain A/canine/Guangdong/04/2014 (H3N2, GD14) were propagated in the allantoic cavities of 10-day-old specific-pathogen-free embryonated hen eggs at 37°C for 72 h. The allantoic fluid was harvested and stored at −80°C.

Rabbit polyclonal antibodies against H3N2 CIV NP were prepared by the Department of Veterinary Clinical Surgery, School of Veterinary Medicine, South China Agricultural University, and stored at −20°C. Mouse anti-FLAG monoclonal antibody was purchased from Sigma (USA, 1:2000 for Western blot and 1:1000 for immunofluorescence staining); goat anti-mouse IgG H&L (Alexa Fluor® 488, 1:2000), goat anti-mouse IgG H&L (Alexa Fluor® 790, 1:10000), and goat anti-rabbit IgG (Alexa Fluor® 680, 1:10000) were purchased from Abcam (UK); and anti-*β*-actin monoclonal antibody was purchased from Cell Signaling Technology (USA, 1:2000).

### 2.2 Cell culture and transfection

Human embryonic kidney cells (HEK 293 T) and Madin-Darby canine kidney (MDCK) cells were maintained in Dulbecco’s Modified Eagle Medium (DMEM) containing 10% fetal bovine serum (FBS). One day after plating, the cells were washed with PBS, and DMEM containing 1% FBS and 1% penicillin–streptomycin was added. The cells were continued to be cultured in an incubator with 5% CO_2_ at 37°C.

### 2.3 Bioinformatics analysis

The sequences of human tetherin, canine tetherin, and tetherin mutational variants (delCT, delCC, delTM, and delGPI) were compared with the nucleotide and amino acid sequences using the BioEdit 7.0.9.1 software (BioEdit, Borland, USA). The nucleotide sequences of canine tetherin and its mutational variants were translated into amino acid sequences using the SnapGene 4.1.6 software (SnapGene, Dotmatics, UK). The amino acid sequences of canine tetherin and its mutational variants were submitted to I-TASSER^1^ for 3D protein structure simulation.

### 2.4 Western blot

The pEF6/Myc-His, wild-type pEF-FLAG-canine-tetherin, and tetherin mutational variants (pEF-FLAG-canine-tetherin-delCT, pEF-FLAG-canine-tetherin-delCC, pEF-FLAG-canine-tetherin-delTM, and pEF-FLAG-canine-tetherin-delGPI) were transiently transfected into the HEK 293 T cells. Furthermore, 24 h post-transfection, the cells were washed twice with PBS, and a RIPA buffer (Epizyme, Shanghai, China) was used to lyse the cells and extract total proteins. Equal amounts of cell lysate were separated using SDS-PAGE on a 12.5% gel, followed by the transfer of the proteins onto a PVDF membrane. The membranes were blocked with 5% (v/v) skim milk powder diluted in PBS at room temperature for 1 h at 37°C. After washing with PBS, the membranes were probed overnight at 4°C with the mouse anti-FLAG monoclonal antibody and rabbit anti-*β*-actin monoclonal antibody, respectively. After antibody recovery and washing with PBS, the PVDF membrane was, respectively, incubated for 1 h at 37°C with goat anti-mouse IgG H&L (Alexa Fluor® 790) and goat anti-rabbit IgG (Alexa Fluor® 680), after being cleaned three times with PBS containing 0.1% Tween-20. Protein bands were visualized with an infrared two-color laser imaging system (Odyssey, Lincoln, NE, USA).

### 2.5 Immunofluorescence staining and confocal microscopy

For microscopy studies, the HEK 293 T cells were evenly spread into 35 mm laser confocal dishes. When the cells grew to 30% ~ 40%, the eukaryotic expression vector of pEF-FLAG-canine-tetherin and tetherin mutational variants were transfected using the transfection reagent lipo8000 (Beyotime, Shanghai, China). After 24 h of transfection, the cells were rinsed with PBS and fixed in 4% (v/v) paraformaldehyde in PBS for 30 min at 4°C. The cells were then rinsed with PBS three times, blocked with the QuickBlock™ blocking buffer (Beyotime, Shanghai, China) for 30 min at room temperature, and incubated with the mouse anti-FLAG monoclonal antibody overnight at 4°C. After washing (containing 0.1% (v/v) Tween-20) the cells three times for 5 min each with PBST, the HEK 293 T cells were incubated with the goat anti-mouse IgG H&L (Alexa Fluor® 488) fluorescence antibody for 1 h at room temperature. After washing with PBST three times, the cells were stained with 4′,6-diamidino-2-phenylindole (DAPI) (Beyotime, Shanghai, China) and examined using laser confocal microscopy.

For each transfection group, 100 fluorescent cells were assessed. The target protein was deemed nuclear if its fluorescence completely or mostly overlapped with the DAPI-stained nuclei. It was considered cytoplasmic if the fluorescence was widespread around the nucleus (outside the DAPI area) with no obvious signal in the cell membrane. Membrane localization was indicated by the fluorescence forming a ring at the cell periphery, with no nuclear overlap.

### 2.6 Detection of cell proliferation ability

The CCK-8 assay was used to determine the proliferation ability of the HEK 293T cells overexpressing canine tetherin and its mutational variants, according to the manufacturer’s instructions (Vazyme, China). The HEK 293 T cells were plated in 96-well plates at a density of 60% ~ 70%, and 100 ng of plasmids was transfected after the cells adhered to the wall. Then, 24 h after transfection, an CCK-8 assay was performed, and the absorbance was measured using a microplate reader at 450 nm. The untreated control was set at 100%, and the survival rate (%) was calculated according to the following formula: [optical density (OD) of the treated cells - OD of blank control/OD of negative control - OD of blank control] × 100%.

### 2.7 One-step growth curve of CIV

The HEK 293T cells were plated in a 24-well plate, infected with H3N2 CIV (MOI = 0.1), and the supernatant was collected every 12 h (12 h ~ 84 h). MDCK cells were plated in 96-well plates and infected with the supernatant. After 48 h of infection, the MDCK cells were washed with PBS. An indirect immunofluorescence assay (IFA) test was performed according to the protocol described in section 2.5. CIV was detected with a rabbit polyclonal antibody against H3N2 CIV NP, and goat anti-rabbit IgG H&L (Alexa Fluor® 488) was used as the secondary antibody. The wells were examined for fluorescence under a fluorescence microscope, and the tissue culture infectious dose 50 (TCID_50_) assay was calculated using the Reed–Muench method.

### 2.8 Viral titer assays

The HEK 293 T cells were transfected with pEF6/Myc-His, wild-type pEF-FLAG-canine-Tetherin, and tetherin mutational variants (pEF-FLAG-canine-tetherin-delCT, pEF-FLAG-canine-tetherin-delCC, pEF-FLAG-canine-tetherin-delTM, and pEF-FLAG-canine-tetherin-delGPI) in 12-well plates, respectively. After 24 h of transfection, the HEK 293 T cells were infected with the H3N2 GD14 CIV strain for 1 h at 37°C with 5% CO_2_. The virus solution was discarded, the cells were washed with PBS, and DMEM containing 1% FBS and 1% penicillin–streptomycin was added. The cells were continued to be cultured in an incubator with 5% CO_2_ at 37°C. Subsequently, the supernatant was collected every 12, 24, and 36 h, and the TCID_50_ of the virus in the supernatant was measured as described above. At 24 h, both cell and supernatant RNA were collected and used for viral RNA (vRNA) extraction. To elucidate the impact of wild-type and mutant canine tetherin on CIV RNA levels, reverse transcription primers specific for CIV-complementary RNA (cRNA), vRNA, and messenger RNA (mRNA) were designed: IAV-cRNA (5′-AACATCCACAGCACTCTGCTGTTCCT-3′), IAV-vRNA(5′-AGTCTTCTAACCGAGGTCGAAACGTA-3′), and IAV-mRNA:Oligo (dT). RT-qPCR was performed using the ChamQ SYBR qPCR Master Mix (Vazyme, Nanjing, China) to quantify the levels of CIV cRNA, vRNA, and mRNA in the infected cells. Relative expression levels were determined using the 2^−∆∆^CT method. The expressing level of the viral M gene was determined using RT-qPCR with specific primers (M-qF: 5′-TGATCCTCTCGTTATTGCCGCAAG-3′, M-qR: 5′-CACTCTGC TGTTCCTGCCGATAC-3′, GAPDH-human-F: 5′-AGATCCCTCC AAAATCAAGTGG-3′, and GAPDH-human-R: 5′-GGCAGAGAT GATGACCCTTTT-3′).

### 2.9 Statistical analysis

All experimental data were analyzed using GraphPad Prism (version 9.0, GraphPad Software, Inc.). For comparisons between the groups, Student’s *t*-tests were used for two-group comparisons, while one-way ANOVA followed by Tukey’s multiple comparisons test was applied for comparisons involving more than two groups. Statistical significance was defined as follows: *p* < 0.05 (^*^), *p* < 0.01 (^**^), and *p* < 0.001 (^***^). Data were presented as mean ± standard deviation (SD) from at least three independent experiments. All experiments included appropriate control groups (e.g., mock-transfected cells or cells transfected with empty vectors) to ensure the validity of the results.

## 3 Results

### 3.1 Tetherin mutational variants construction strategy and bioinformatics analysis

**Figure 1:**
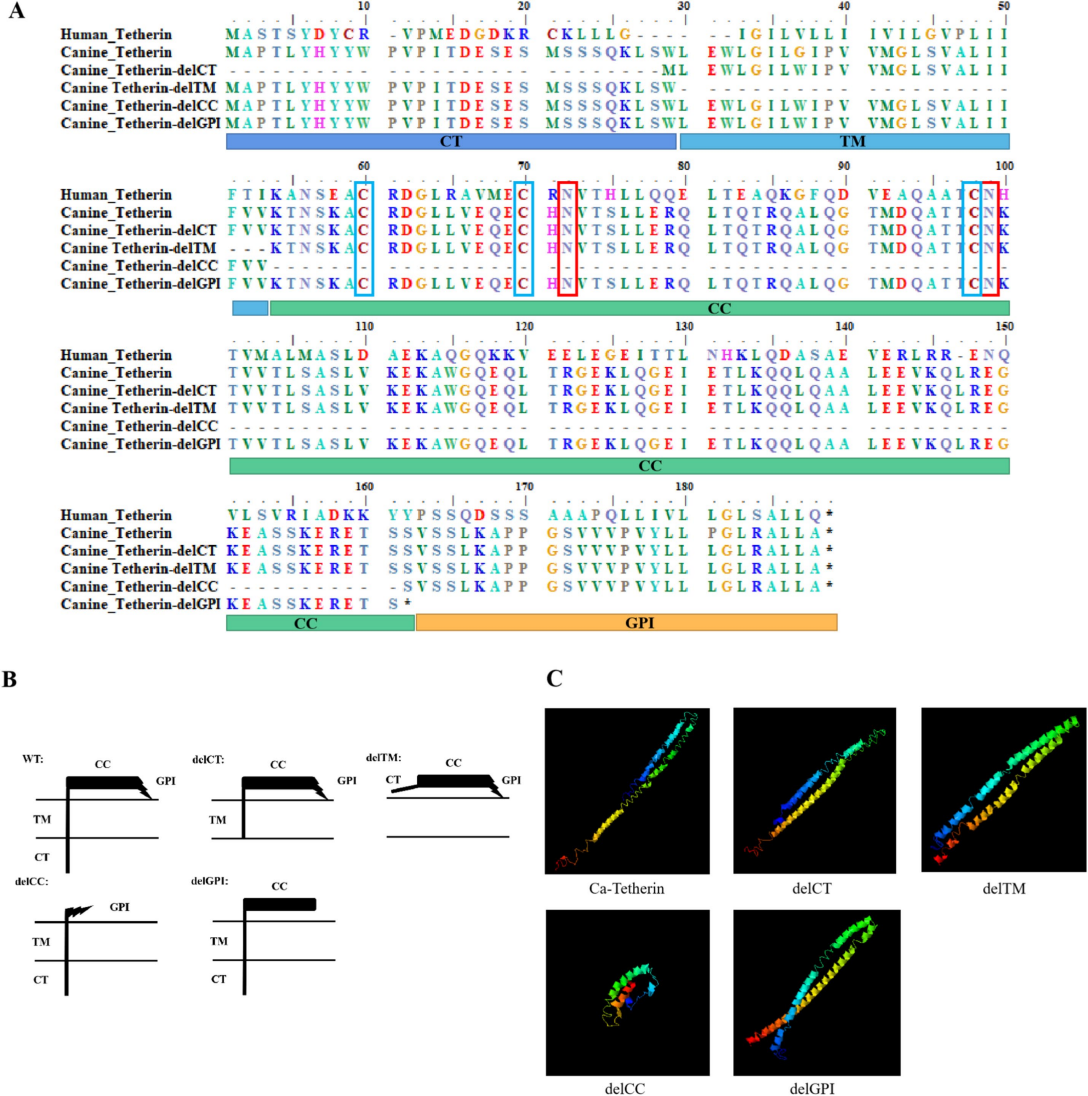
Sequence alignment, schematic representation of tetherin mutational variants, and 3D structures of canine tetherin and its mutational variants. **(A)** Amino acid sequence alignment of human tetherin, canine tetherin, and mutational variants. The glycosylation sites of canine tetherin (N72 and N99) and human tetherin (N65 and N92) are marked with red boxes, while the three conserved cysteine residues of canine tetherin (C60, C70, and C99) and human tetherin (C53, C61, and C91) are marked with blue boxes. **(B)** Schematic representation of canine tetherin and its mutational variants, including the deletion mutations at the N-terminal cytoplasmic tail (delCT), ectodomain coiled-coil region (delCC), or membrane anchors (delGPI and delTM). **(C)** Simulation 3D structures of canine tetherin and its mutational variants. All protein structures in the PDB database were analyzed using the SPICKER program of I-TASSER, and the five most probable structural cluster models are reported. The feasibility of each model is quantified using a c-score (−5, 2), with a higher c-score indicating greater feasibility of the model. The most reliable models were selected to simulate the 3D structures of canine tetherin and its mutational variants.

Tetherin is a homodimeric, lipid raft-associated type II integral membrane glycoprotein. The amino acid sequence alignment results of canine tetherin and human tetherin are shown in Figure 1A, with glycosylation sites of canine tetherin (N72 and N99) and human tetherin (N65 and N92) being conserved. Moreover, there are also three conserved cysteine residues in canine tetherin (C60, C70, and C99), and these residues bind to tetherin in the form of a dimer. Based on the amino acid alignment results, we divided the structural domains of canine tetherin. To determine how the structural domains affect the ability of canine tetherin to inhibit CIV, we generated canine tetherin mutational variants (delCT, delCC, delTM, and delGPI), as depicted in Figure 1B.

The 3D structures of canine tetherin and its mutational variants were simulated using the I-TASSER software, with the most reliable model serving as the 3D structural model of canine tetherin (Figure 1C). The results showed that the spatial structures of the canine tetherin mutational variants delCT, delTM, and delGPI were similar to wild-type canine tetherin, but the canine tetherin mutational variant delCC exhibited a different spatial structure.

### 3.2 Expression of canine tetherin and its mutational variants

**Figure 2:**
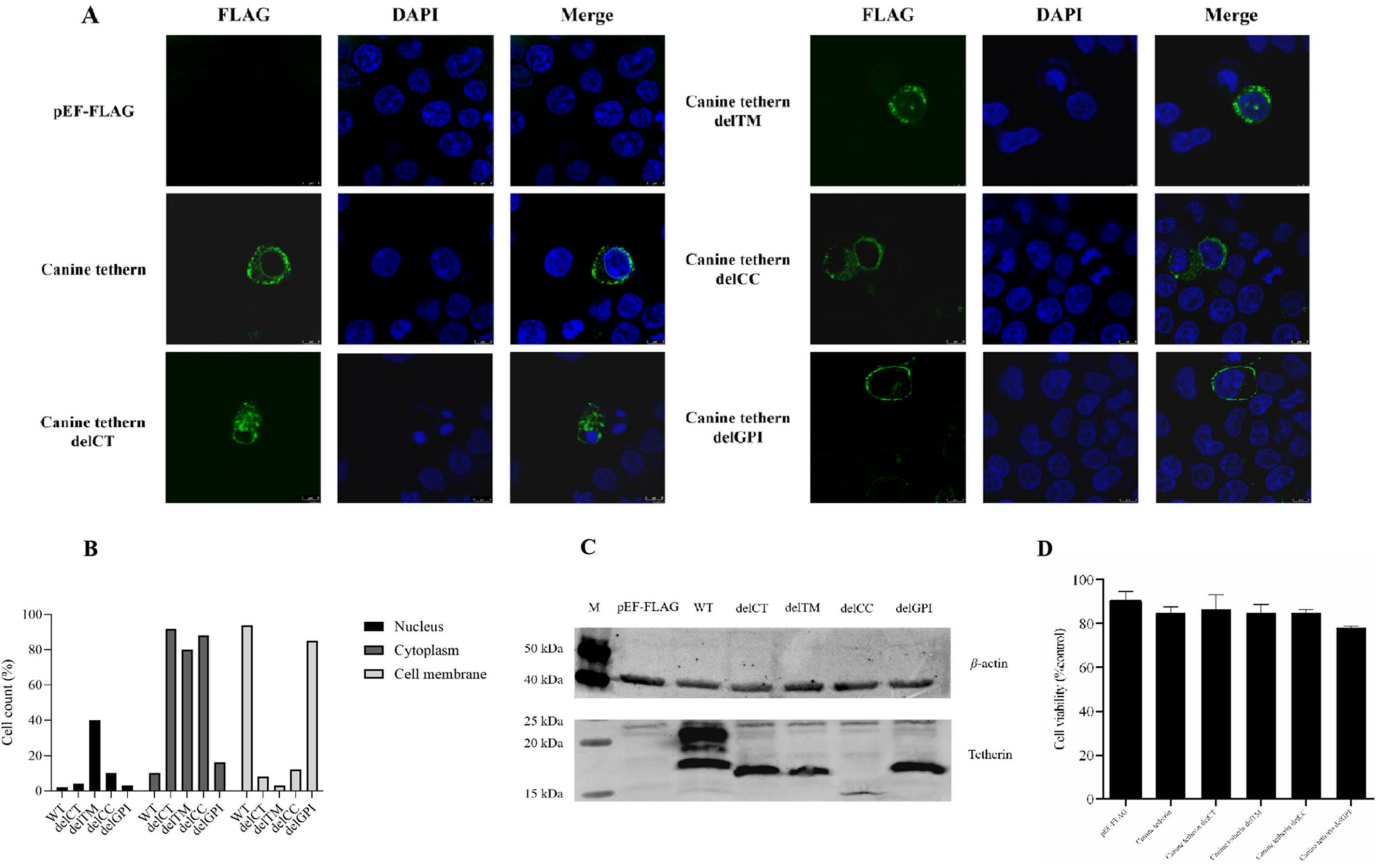
Subcellular localization of canine tetherin and its mutational variants. **(A)** HEK 293 T cells were transfected with FLAG-tagged canine tetherin and its mutational variant expression constructs. After 24 h, the cells were stained with an anti-FLAG antibody (green), and the nucleus was stained with DAPI (blue). Then, the cells were analyzed using confocal microscopy. **(B)** For each transfection group, 100 fluorescent cells were selected for observation, and the number of cells in which the fluorescent signals of the target protein were mainly distributed in the nucleus, cytoplasm, or cell membrane was counted. **(C)** HEK293T cells were transfected with 2.5 μg of each tetherin mutant. After 24 h of transfection, the cells were lysed with a RIPA buffer. A total of 30 ng of total protein was used for SDS-PAGE. Lane M, protein marker; lane 1, pEF-FLAG; lane 2, canine tetherin; lane 3, canine tetherin delCT, lane 4, canine tetherin delTM; lane 5, canine tetherin delCC; and lane 6, canine tetherin delGPI. **(D)** Cell viability was detected using a cell proliferation assay. ^*^p < 0.05, ^**^p < 0.01, and ^***^p > 0.05.

The eukaryotic expression plasmids pEF6/Myc-His, wild-type pEF-FLAG-canine-tetherin, and canine tetherin mutational variants (pEF-FLAG-canine-tetherin-delCT, pEF-FLAG-canine-tetherin-delCC, pEF-FLAG-canine-tetherin-delTM, and pEF-FLAG-canine-tetherin-delGPI) were transiently transfected into the HEK 293T cells. After 24 h, the protein expression was identified using Western blot (Figure 2C). The Western blot results for canine tetherin showed three bands, with sizes ranging from 15 kDa to 35 kDa. In contrast, the mutational variants showed single bands, with sizes ranging from 9 kDa to 35 kDa. Notably, the delCC mutant exhibited poor expression. Cell viability assays using the CCK8 method showed no cytotoxic effects of canine tetherin or its mutational variants on the HEK 293 T cells (*p* > 0.05) (Figure 2D).

### 3.3 Subcellular localization of canine tetherin and its mutational variants

To understand whether the deletion of a domain in canine tetherin affects its cellular localization, the HEK 293 T cells were transiently transfected with canine tetherin and four canine tetherin mutational variants constructs, each with an N-terminal FLAG tag. The results, shown in Figures 2A,B, demonstrated that wild-type canine tetherin and the delCT, delCC, and delGPI mutational variants were expressed at the plasma membrane and in intracellular locations. However, the delTM mutational variant lacked the ability to localize to the cell membrane and was only present in the cytoplasm.

### 3.4 Canine tetherin and its mutational variants restrict the replication of CIV

**Figure 3:**
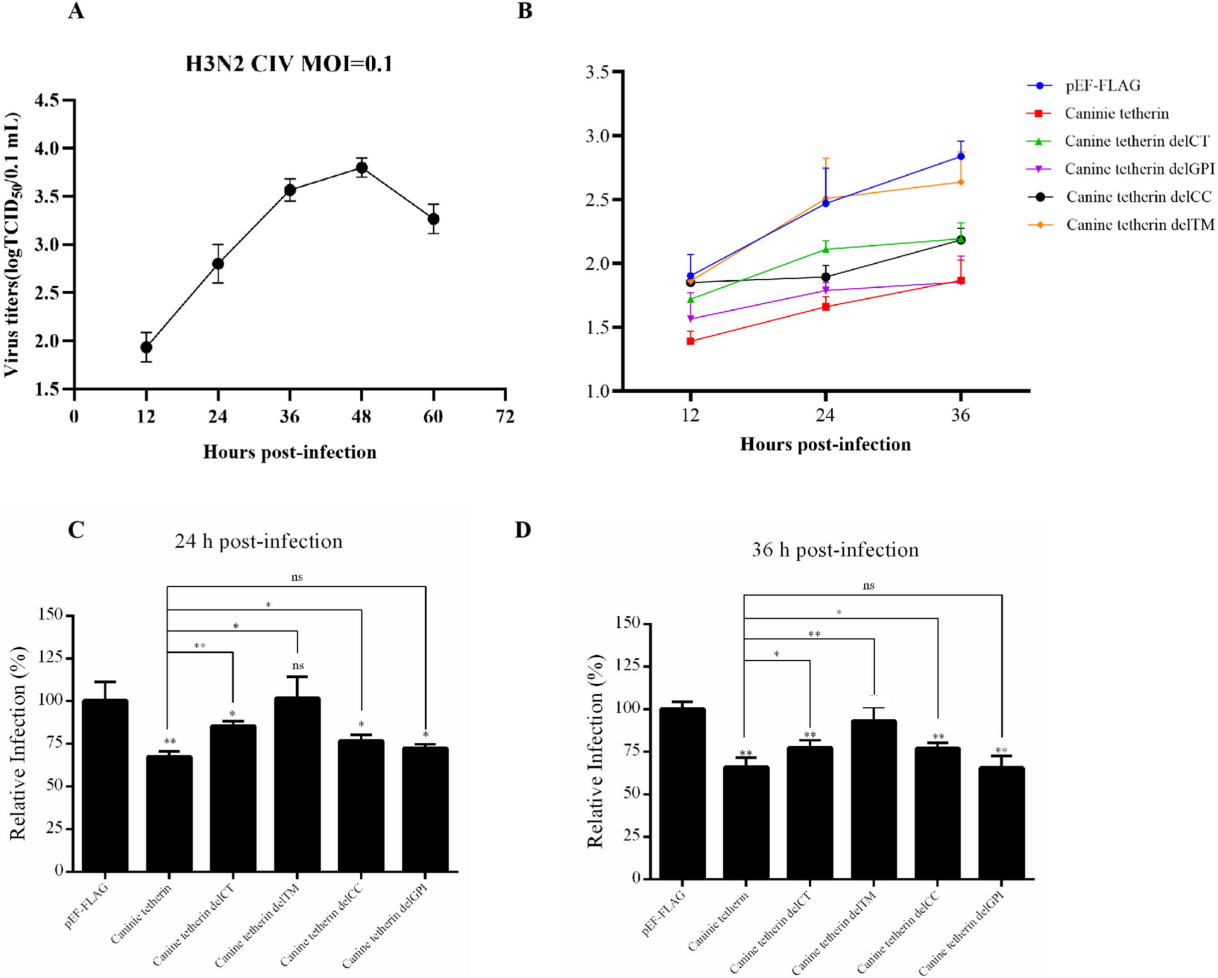
Effects of wild-type canine tetherin and its mutational variants on CIV release. **(A)** One-step growth curve of CIV in HEK 293 T cells. The HEK 293T cells were infected with H3N2 CIV (GD14) at an MOI of 0.1. Viral titers in the supernatant at each time point (12 h ~ 60 h) were determined using the TCID_50_ assay in MDCK cells. **(B)** Canine tetherin restricts the release of H3N2 CIV. HEK 293 T cells were transfected with 1,000 ng of each plasmid: pEF6/Myc-His, wild-type pEF-FLAG-Canine-Tetherin, and tetherin mutational variants pEF-FLAG-Canine-Tetherin-delCT, pEF-FLAG-Canine-Tetherin-delCC, pEF-FLAG-Canine-Tetherin-delTM, and pEF-FLAG-Canine-Tetherin-delGPI. Moreover, 24 h post-transfection, the cells were infected with H3N2 CIV GD/2014 at an MOI of 0.1. Viral titers in the supernatant from each time point were determined using the TCID_50_ assay. **(C,D)** The TCID_50_ assay results at 24 h and 36 h (^*^p < 0.05; ^**^p < 0.01; ns: no significance *p* > 0.05).

To determine the replication kinetics of H3N2 CIV (GD14) in HEK 293 T cells, a one-step growth curve was established (Figure 3A). The viral titer in the supernatant peaked at 48 h post-infection and reached a plateau at 60 h. The HEK 293 T cells were transfected with wild-type canine tetherin and its mutational variants, followed by infection with H3N2 CIV (MOI = 0.1). The viral titers in the supernatant were measured at different time points using the TCID_50_ assay (Figures 3B–D). The results showed that deletion of the TM region (delTM) abolished the ability of canine tetherin to restrict CIV replication (*p* > 0.05). Deletion of the CT (delCT) or CC (delCC) domains reduced the antiviral activity of canine tetherin, but the protein still retained some ability to restrict CIV replication (24 h: *p* < 0.05 or *p* < 0.01; 36 h: *p* < 0.01 or *p* < 0.05). Notably, deletion of the GPI domain (delGPI) did not affect the antiviral activity of canine tetherin.

**Figure 4:**
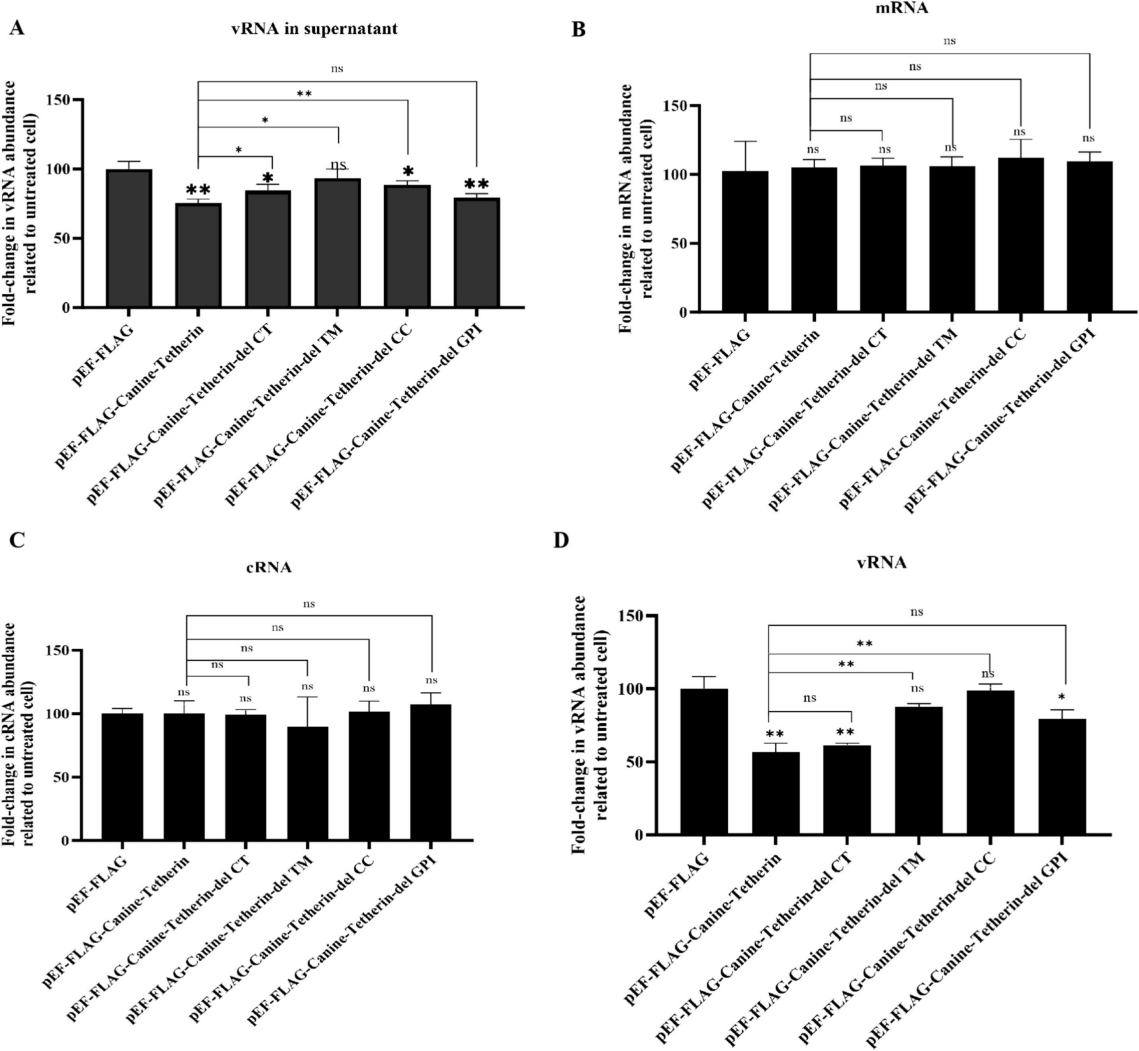
Effects of wild-type canine tetherin and its mutational variants on CIV RNA levels. **(A)** HEK 293 T cells were transfected with 1 μg of each plasmid: pEF6/Myc-His, wild-type pEF-FLAG-Canine-Tetherin, and tetherin mutational variants pEF-FLAG-Canine-Tetherin-delCT, pEF-FLAG-Canine-Tetherin-delCC, pEF-FLAG-Canine-Tetherin-delTM, and pEF-FLAG-Canine-Tetherin-delGPI. Moreover, 24 h post-transfection, the cells were infected with H3N2 CIV GD/2014 at an MOI of 0.1. Viral RNA in the supernatant and viral RNA, cRNA, and mRNA in the cells were determined using RT-qPCR. (^*^p < 0.05; ^**^p < 0.01; ns: no significance *p* > 0.05).

To further investigate the effect of tetherin on viral RNA synthesis, viral RNA (vRNA), complementary RNA (cRNA), and messenger RNA (mRNA) levels were measured using RT-qPCR in the cells infected with CIV (MOI = 0.1) (Figure 4). The results showed a significant decrease in the vRNA levels in both the supernatants and cells upon overexpression of tetherin, while the mRNA and cRNA levels remained largely unaffected.

## 4 Discussion

Influenza viruses are zoonotic pathogens that infect a variety of host species, posing a serious threat to global public health. As a “mixing vessel” for influenza viruses, antiviral research on canine natural immune factors is crucial for the prevention and control of influenza virus outbreaks. Recent research has shown that canine tetherin can restrict CIV. However, the main functional domain responsible for its antiviral activity is still unclear. Therefore, in this study, we divided the structural domains of canine tetherin by comparing its amino acid sequences with that of human tetherin and analyzed the effects of each functional domain on cellular localization and the restriction of CIV replication.

Due to its glycosylation pattern, the Western blot results of canine tetherin showed three bands, with sizes ranging from 15 kDa to 35 kDa (Figure 2C). This is consistent with previous research on human tetherin and canine tetherin (5, 6). The presence of multiple bands in the Western blot analysis suggests that canine tetherin undergoes post-translational modifications, particularly N-linked glycosylation. Glycosylation is a common modification for membrane proteins and can influence protein folding, stability, and function. In the case of tetherin, glycosylation may affect its ability to restrict viral release by altering its conformation or membrane localization (23, 24).

To further investigate the glycosylation status of canine tetherin, we performed PNGase F treatment on the cell lysates. PNGase F is an enzyme that specifically removes N-linked glycans from glycoproteins, thereby reducing their apparent molecular weight on SDS-PAGE. After the PNGase F treatment, the multiple bands observed for wild-type canine tetherin were resolved into a single band with a lower molecular weight (approximately 15 kDa), confirming that the additional bands observed in the untreated samples were due to N-linked glycosylation (Supplementary Figure 1). This finding aligns with those of studies on human tetherin, which also demonstrated the importance of glycosylation in its antiviral function (25).

However, the Western blot results for the canine tetherin mutational variants showed only a single band, with sizes ranging from 9 kDa to 35 kDa, suggesting that domain deletion may affect the glycosylation modification pattern of canine tetherin (Figure 2C). It was noted that the delCC deletion mutant was poorly expressed, which is consistent with previous reports on human tetherin (23, 26).

Tetherin is a homodimeric, lipid raft-associated type II membrane glycoprotein, and it forms stable cysteine-linked dimers and is modified by N-linked glycosylation. After comparing the amino acid sequence with human tetherin, we found that the N-linked glycosylation sites (N72 and N99) and cysteine residues (C60, C70, and C98) of canine tetherin were all conserved and located in the extracellular domain of canine tetherin (CC) (23, 24). Similar to human tetherin, the Western blot results for canine tetherin showed three bands due to its N-linked glycosylation. However, when the other domains were (CT, TM, and GPI) deleted, the Western blot results only showed one band. This result indicates that the conformation of the protein changes, although its N-linked glycosylation sites are not affected when its domains are deleted. However, whether these N-linked glycosylation sites and cysteine residues are important for the antiviral activity of canine tetherin still requires further research (23, 24).

In a study of the subcellular localization of tetherin in cells, previous confocal laser experiments on human tetherin found that both wild-type and human tetherin mutational variants (delCT, delCC, delTM, and delGPI) were expressed at the plasma membrane and in intracellular locations (24). However, in this study, we found that the delTM mutational variant of canine tetherin was only present in the cytoplasm (Figure 2). This result indicates that the cell membrane localization signal of canine tetherin mainly exists in the TM region and that it may be different from human tetherin, which has multiple membrane localization signals.

The TM domain of human tetherin (amino acid positions 22 ~ 43) is a short, one-way *α*-helix that anchors the molecule at the plasma membrane, while the GPI anchor is located in the C-terminal region of the protein. These two membrane anchors partially determine the antiviral function of human tetherin (24). In this study, we found that the delTM mutational variant of canine tetherin, which lacks the N-terminal transmembrane domain, did not block CIV release (Figures 3B–D). The DelCT and delCC domains reduced the antiviral activity of canine tetherin, but the protein still retained some ability to restrict CIV replication (Figures 3B–D). However, deletion of the delGPI domain did not affect the antiviral activity of canine tetherin, which is inconsistent with previous studies on human tetherin (24). The reason for this difference is unclear, and further research is needed to analyze the mechanism behind canine tetherin’s antiviral replication. Combined with the subcellular localization results of canine tetherin in cells (Figure 4), we found that the antiviral activity of canine tetherin may be related to its subcellular localization in cells.

In conclusion, our findings demonstrate that understanding the structure and antiviral function of canine tetherin may advance the study of cross-host transmission of influenza viruses and ultimately contribute to the development of antiviral therapeutic strategies against influenza virus infections. To further elucidate the multifaceted role of canine tetherin in antiviral defense, future investigations should focus on characterizing its activity against a broader spectrum of canine-infecting viruses. In addition, efforts should be directed toward identifying potential viral proteins within the influenza virus repertoire that may antagonize the antiviral restriction imposed by canine tetherin, thereby facilitating viral persistence and infection.

## Data Availability

The datasets presented in this study can be found in online repositories. The names of the repository/repositories and accession number(s) can be found in the article/supplementary material.

## Footnotes

1
https://zhanglab.ccmb.med.umich.edu/I-TASSER/
