# Correction: Identification of the functional domains of canine tetherin in antiviral activity against canine influenza virus

**DOI:** 10.3389/fvets.2026.1888319

**Published:** 2026-06-15

**Authors:** Jiajun Ou, Yixin Dai, Yujie Jiang, Lingzhi Dai, Liang Xu, Gang Lu, Gaoming Lou, Shoujun Li

**Affiliations:** 1 College of Veterinary Medicine, South China Agricultural University, Guangzhou, China; 2 Henry Fok School of Biology and Agriculture, Shaoguan University, Shaoguan, China; 3 Guangdong Provincial Key Laboratory of Utilization and Conservation of Food and Medicinal Resources in Northern Region, Shaoguan University, Shaoguan, China

**Keywords:** tetherin, canine influenza virus, antiviral activity, functional domain, influenza A virus

In the original article, there was an error. The information for one research grant (Guangdong Provincial Natural Science Foundation) was erroneously omitted.

The correct **Funding** statement reads:

The author(s) declare that financial support was received for the research and/or publication of this article. This work was supported by the Natural Science Foundation of China (32172826) and Guangdong Provincial Natural Science Foundation (2023A1515012950).

The original version of this article has been updated.

